# Lysozyme Amyloid for Synthetic RNA Delivery

**DOI:** 10.3390/pharmaceutics17091094

**Published:** 2025-08-22

**Authors:** Benjamin Beluzo, Maytham Ismail, Sergei Chuikov, Venkateshwar G. Keshamouni, Mathumai Kanapathipillai

**Affiliations:** 1 Department of Mechanical Engineering, University of Michigan-Dearborn, Dearborn, MI 48128, USA; bbeluzo@umich.edu (B.B.); mgismail@umich.edu (M.I.); 2 Division of Pulmonary and Critical Care Medicine, Department of Internal Medicine, University of Michigan, Ann Arbor, MI 48109, USA; schuikov@umich.edu (S.C.); vkeshamo@med.umich.edu (V.G.K.); 3 LTC Charles S. Kettles VA Medical Center, Research Service (151), Ann Arbor, MI 48109, USA

**Keywords:** amyloid aggregates, lysozyme, RNA delivery

## Abstract

**Background/Objectives**: Lysozyme-based amyloid aggregates offer a promising platform for RNA delivery due to their stability, cationic nature, biocompatibility, and ability to form well-defined structures. In this study, we evaluated their potential as drug carriers, focusing on the delivery of polyinosinic–polycytidylic acid (Poly(I:C)), an immunostimulatory synthetic RNA. To validate RNA delivery capability and rule out the possibility that observed effects arose from the lysozyme–Poly(I:C) complex itself, small interfering RNA (siRNA) was also used to verify that the successful delivery of intact and functional RNA was the cause of the observed effects. **Methods**: The aggregates were characterized by particle size, zeta potential, morphology, and RNA encapsulation efficiency. **Results**: In vitro studies using RAW 264.7 macrophage-like cells demonstrated that Poly(I:C)-loaded aggregates improved RNA uptake and triggered significant immune activation without inducing toxicity. To further confirm the potential of lysozyme amyloids in RNA delivery, GFP siRNA-loaded aggregates were evaluated in A549-GFP cells. A notable decrease in GFP expression, confirmed through confocal microscopy and flow cytometry, confirmed successful intracellular delivery. **Conclusions**: These results highlight the potential of lysozyme amyloids as non-viral vectors for RNA delivery, with promising applications in immunotherapy.

## 1. Introduction

The rapid advancement of RNA-based therapeutics has intensified the need for delivery systems that are efficient, biocompatible, and capable of protecting nucleic acids from degradation. Therapies involving small interfering RNA (siRNA), messenger RNA (mRNA), and synthetic analogs such as polyinosinic–polycytidylic acid (Poly (I:C)) are showing significant promise for applications in gene regulation, immunotherapy, and vaccine development [1,2,3]. However, these nucleic acids are often unstable in physiological environments, prone to enzymatic degradation, and face barriers to cellular uptake, necessitating the development of safe and effective delivery platforms [4,5,6].

One emerging solution is the use of amyloid-like protein aggregates, which form stable nanostructures capable of encapsulating and protecting therapeutic molecules [7,8,9]. Though historically associated with neurodegenerative disease, functional amyloids are increasingly recognized for their roles in physiological processes and utility in biomedical engineering [10,11]. Their mechanical stability, ability to self-assemble, and compatibility with biologics make them attractive candidates for drug delivery, immunomodulation, and tissue engineering [12,13,14]. Notably, their potential to enhance drug stability and facilitate cellular delivery through encapsulation and protection mechanisms has positioned amyloid materials as a promising alternative to conventional carriers [15,16,17].

Among amyloid-forming proteins, lysozyme has gained attention due to its favorable properties. Lysozyme is a cationic protein with well-documented amyloidogenic behavior under acidic or denaturing conditions [18,19,20]. It is naturally expressed by innate immune cells such as macrophages, neutrophils, and monocytes, and plays a key role in antimicrobial defense and inflammation [21,22]. The cationic charge of lysozyme fibrils enables strong electrostatic interactions with negatively charged nucleic acids [23,24]. Additionally, lysozyme amyloids are known to be biocompatible and have been explored for applications ranging from antimicrobial coatings to drug depots and injectable hydrogels [25,26]. This immunological relevance, structural integrity, and strong oligonucleotide-binding affinity suggest lysozyme amyloids are particularly well-suited for synthetic RNA delivery.

Poly (I:C) is a synthetic double-stranded RNA analog that mimics viral RNA and activates Toll-like receptor 3 (TLR3) signaling pathways [27,28]. This activation leads to robust type I interferon responses, cytokine secretion, and immune cell recruitment, making Poly (I:C) a widely used immunostimulant in antiviral and vaccine applications [29,30]. However, its clinical utility is limited by rapid degradation and nonspecific distribution [31,32]. To overcome these limitations, combining Poly (I:C) with protective delivery depots like lysozyme amyloids may enhance stability, improve targeting, and amplify immunogenicity without introducing significant cytotoxicity.

It was also important to verify that the lysozyme delivery system was successfully delivering functional Poly (I:C) or synthetic RNA and not an emergent property of complexing them together; hence the delivery of an siRNA validation control utilizing lysozyme amyloids could further the potential of the system in RNA delivery. siRNA is a double-stranded RNA molecule that engages the RNA interference (RNAi) pathway to selectively silence gene expression. Once internalized, the siRNA duplex is incorporated into the RNA-induced silencing complex (RISC), where the passenger strand is degraded and the guide strand directs RISC to complementary mRNA for cleavage and degradation [33,34]. This leads to decreased expression of the target protein; in this study the delivery of GFP-siRNA is employed. 

In this study, we investigate lysozyme amyloid aggregates as a delivery system for synthetic RNAs. We evaluate their physicochemical characteristics, RNA encapsulation efficiency, and biological effects in immune-relevant cell models. Using Poly (I:C) in RAW 264.7 macrophage-like cells, we assess cellular uptake and immunostimulatory activity. Additionally, we validate RNA delivery by using GFP-targeting siRNA in a stable A549-GFP reporter cell line. 

## 2. Materials and Methods

### 2.1. Materials

Lysozyme from chicken egg whites and Thioflavin-T (ThT) dye were sourced from Millipore Sigma (Milwaukee, WI, USA). Polyinosinic–polycytidylic acid sodium salt (Poly (I:C)) was attained through Sigma Aldrich (St. Louis, MO, USA), while the high-molecular-weight Rhodamine-labeled Poly (I:C) (HMW) was obtained from InvivoGen (San Diego, CA, USA). BCA Protein Assay Kit, various cell culture reagents, fetal bovine serum, and antibiotic–antimycotic solution were purchased from Thermo Fisher Scientific (Waltham, MA, USA). The Griess Reagent was provided by Enzo Life Sciences (Farmingdale, NY, USA), and MitoROS 580 was obtained from AAT Bioquest (Pleasanton, CA, USA). Additionally, AlamarBlue Cell Viability Reagent and Invitrogen Silencer GFP (eGFP) siRNA were purchased from Invitrogen (Waltham, MA, USA).

### 2.2. Protein Aggregate Formation

Lysozyme protein was prepared in 150 mM NaCl buffer (pH 2) to achieve a concentration of 5 mg/mL. Amyloid formation was initiated at 60 °C using a thermomixer. For cellular efficacy and formulation studies, lysozyme solutions (5 mg/mL) were mixed with high-molecular-weight Poly (I:C) (10 mg/mL) at a 2:1 mass ratio (Poly (I:C)/lysozyme) prior to incubation to form co-aggregates. After amyloid formation, all protein aggregates were concentrated via centrifugation at 15,000× *g* at 4 °C for 30 min, then resuspended with 0.01 M PBS (pH 7.4). For the siRNA transfection study, after centrifugation, (eGFP) siRNA (0.5 μg) was mixed with lysozyme to form the lysozyme–siRNA formulation. The protein concentration of amyloid samples after resuspension was quantified with the BCA Protein Assay Kit (Thermo Fisher Scientific). Samples and calibration curves were prepared according to the manufacturer’s protocol.

### 2.3. Thioflavin T (ThT) Fluorescence

Amyloid aggregation was verified using ThT fluorescence, a widely recognized method for detecting amyloid structures [12]. For each reading, 5 µL of the sample was combined with 100 µL of 50 µM ThT prepared in 50 mM Tris buffer (pH 8). Fluorescence was measured at 30 min, 24 h, and 48 h using 440 nm excitation and 482 nm emission wavelengths. Measurements were performed using a SpectraMax M3 spectrophotometer (Molecular Devices, San Jose, CA, USA), and each experiment was repeated at least three times independently.

### 2.4. Size, Zeta Potential, Morphology, and Protein Absorption

Transmission Electron Microscopy (TEM) imaging and dynamic light scattering (DLS) measurements were performed to assess size and morphology of the aggregates. Samples were spotted on a 400-mesh copper grid and stained with 2% phosphotungstic acid (pH 7.4). The JEOL TEM microscope (Peabody, MA, USA) at the Electron Microscopy Facility of the University of Michigan Medical School in Ann Arbor was used. For the DLS measurement, 5 µL of the samples was diluted with 50 µL PBS buffer and the size was measured using Malvern Zetasizer (Westborough, MA, USA). The surface charge of the aggregates was also measured by Malvern Zetasizer instrument. A total of 70 µL of each sample was mixed with 700 µL of distilled water, loaded into disposable capillary cells, and zetapotential was measured. For the protein absorption, the aggregate formulations (Lyz, Lyz-Poly (I:C), Lyz-GFP siRNA) were incubated with FITC-Albumin at a 10:1 weight ratio for 1 h at 37 °C. The aggregates were then centrifuged, and protein absorption was measured by FITC fluorescence at 495/520 nm excitation/emission. 

### 2.5. Polyinosinic–Polycytidylic (Poly(I:C)) Encapsulation and Release Study 

Encapsulation and release studies were conducted to determine Poly (I:C) concentrations for efficacy experiments. First a Poly (I:C) fluorescence vs. concentration standard calibration curve using Rhodamine-labeled HMW Poly (I:C) was obtained. Poly (I:C) concentrations in the range of 0–100 µg/mL were used, and fluorescence measurements were performed at 546/576 nm excitation and emission wavelengths, as reported by the manufacturer for Poly (I:C) HMW Rhodamine (InvivoGen, San Diego, CA, USA). Next, the amount of Poly(I:C) in the amyloid formulation was calculated using the standard calibration curve (linear equation: y = 0.484x – 0.6942). 

Encapsulation efficiency was determined by calculating the ratio of the initial amount of Poly (I:C) used in the formulation to the amount present after resuspension. To determine the degree of Poly (I:C) release, aggregated samples were incubated at 37 °C in PBS buffer, and the amount of Poly (I:C) released at 24 and 48 h were calculated using their respective calibration curves. The Poly (I:C) release percentage was calculated as the ratio of Poly (I:C) released at each time point to the total amount present at initial time zero.

### 2.6. Cellular Uptake and Silencer GFP (eGFP) siRNA Transfection Study

First, cellular uptake of the amyloid aggregates was investigated using flow cytometry. The aggregates were prepared with Coumarin and Poly (I:C) HMW Rhodamine for the flow cytometry studies. RAW 264.7 cells were obtained from ATCC and cultured in DMEM supplemented with 10% fetal bovine serum (FBS) and 1% antibiotic–antimycotic according to protocol. For flow cytometry analysis, RAW 264.7 cells were cultured at 50,000 cells/well in a 24-well plate. After 24 h, cells were treated with lysozyme alone and Coumarin-dyed Lyz-Poly (I:C) HMW Rhodamine. Cells were then trypsinized, resuspended in PBS buffer, and kept on ice. Uptake was assessed using flow cytometry within 30 min of trypsinization on an Attune flow cytometer. The flow cytometry test flow conditions were set at 10,000 events (cell number). 

Next, the transfection study was investigated using both flow cytometry, confocal microscopy, and a GFP quantification kit (Abcam, Waltham, MA, USA). A549 GFP cells (developed in Dr. Keshamouni’s lab) were used for this study. Cells were cultured in RPMI medium, 10% fetal bovine serum, and 1% antibiotic–antimycotic, and seeded at 50,000 cells/well in a 24-well plate. After 24 h, cells in each well were treated with 5 mg/mL Lyz-GFP siRNA containing 0.5 µg of siRNA. After 24 h the cells were analyzed and measured using the Attune Nxt Flow Cytometer from Thermo Fisher (Waltham, MA, USA). For confocal imaging, a similar cell culture procedure was followed. After 24 h the cells were washed three times with a sterile PBS buffer. Cells were then fixed with 4% paraformaldehyde (PFA) for 15 min and washed again three times with PBS. Imaging was performed using a Leica confocal microscope (Deerfield, IL, USA) in the lab. As for the GFP quantification, a similar cell culture procedure was followed. After 24 h the cells were homogenized and the GFP content was measured using a standard curve generated with standard GFP amounts at 488/507 nm excitation/emission according to the manufacture’s protocol.

### 2.7. Cellular Toxicity 

To assess whether the aggregates induced any adverse effects in cells, an AlamarBlue assay was performed. RAW 264.7 cells were seeded at a density of 20,000 cells/well in a 96-well plate. For cellular efficacy studies, lysozyme solutions (5 mg/mL) were mixed with high-molecular-weight Poly (I:C) (10 mg/mL) at a 2:1 mass ratio (Poly (I:C)/lysozyme) to form Lyz-Poly (I:C) formulations. After 24 h, cells were treated with lysozyme, encapsulated Poly (I:C), and free Poly (I:C) at 40 and 80 µg/mL and then incubated for an additional 24 h. For the toxicity assessment, the AlamarBlue assay was performed according to the manufacturer’s protocol. Cell viability was measured by assessing metabolic activity of the cells at 570/590 nm excitation and emission. Experiments were repeated at least three times.

### 2.8. NO Production and Mitochondrial ROS (MitoROS) Assay 

To test whether the protein aggregates induced any immune response, Griess reagent assay and MitoROS assay were performed. Griess reagent assay is widely used to measure nitric oxide (NO) production in cells, while MitoROS is a key indicator of mitochondrial reactive oxygen species, which are associated with inflammatory response and innate immune response. RAW 264.7 cells were seeded at a density of 20,000 cells/well in a 96-well plate. After 24 h, cells were treated with lysozyme, encapsulated Poly (I:C), and free at 40 and 80 µg/mL. For both NO assay and MitoROS assay, cells were incubated for an additional 24 h. 

Nitric oxide (NO) production was measured using the Griess reagent. An equal volume of the reagent was added to each well, and the plate was incubated in the dark at room temperature for 15 min. Absorbance was then recorded at 540 nm. To evaluate mitochondrial reactive oxygen species (ROS), the MitoROS 580 assay kit from AAT Bioquest was used. A stock solution of MitoROS 580 was first prepared and then diluted at a ratio of 1 part to 500 parts sterile PBS to make the working solution. Cells were incubated with the working solution for 30 min, followed by three gentle washes with sterile PBS. Finally, 100 µL of PBS was added to each well, and fluorescence was measured at an excitation wavelength of 500 nm and emission at 582 nm. All experiments related to NO and mitochondrial ROS were carried out in at least three independent repeats.

### 2.9. Statistical Analysis

Data from all experiments, conducted in three independent replicates, are reported as the mean ± SEM. Differences among groups were analyzed using a one-way ANOVA, and pairwise comparisons were performed using Tukey’s (HSD) test. Statistical significance was considered as the following threshold: * *p* < 0.05, ** *p* < 0.01, *** *p* < 0.001, and **** *p* < 0.0001.

## 3. Results

**Figure 1:**
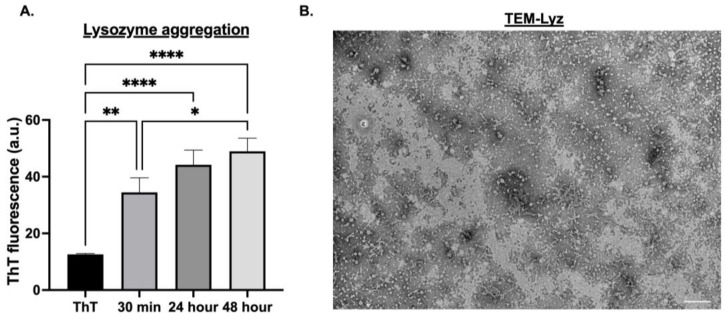
Characterization of lysozyme amyloid aggregates. (**A**) Thioflavin-T (ThT) fluorescence assay was used to verify lysozyme amyloid aggregation at 30 min, 24 h, 48 h. A significant increase in fluorescence was observed at 24 h and 48 h compared to 30 min and with ThT alone. Data are shown as mean ± SEM (*n* = 3). Statistical significance is indicated as * *p* < 0.05, ** *p* < 0.01, **** *p* < 0.0001. (**B**) Transmission electron microscopy (TEM) images of 24 h aggregates revealed low-density, short fibrillar structures ranging from 50 to 100 nm, consistent with amyloid morphology. Scale bar: 200 nm.

Lysozyme (Lyz) amyloid aggregates were first characterized to confirm their formation and suitability as delivery depots. ThT measurements at 440/482 nm excitation/emission were taken to confirm amyloid formation and determine aggregation over 30 min, 24 h, and 48 h. TEM images were performed to determine size and morphology. Additionally, zeta potential was analyzed to determine surface charge and aggregation behavior (Figure 1A,B, Table 1). A significant increase in aggregation was observed at 24 and 48 h compared to 30 min (*p* < 0.0001), with 30 min showing the lowest ThT fluorescence signal (*p* < 0.01, Figure 1A). No significant difference was observed between 24 and 48 h, indicating aggregation plateaued after 24 h. Thus, all subsequent formulations used a 24 h aggregation protocol (Figure 1A). TEM imaging confirmed the formation of low-density, short fibrillar aggregates ranging from 50 to 100 nm (Figure 1B). However, the size measurements by DLS exhibited a slightly larger average size of 183.77 ± 46.73 nm, maybe due to the fibrillar morphology and aggregation of the fibrillar structures. Zeta potential analysis of Lyz aggregates revealed a surface charge of 18.433 ± 0.578 mV (Table 1), confirming the cationic nature critical for electrostatic binding of anionic RNA. Overall, ThT fluorescence, TEM, DLS, and zeta potential confirmed the amyloid-like structure and charge profile of Lyz aggregates suitable for RNA delivery.

**Figure 2:**
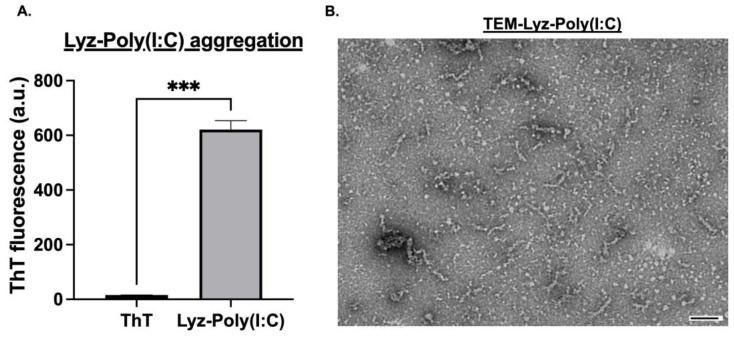
Characterization of lysozyme–poly(I:C) aggregates. (**A**) Thioflavin-T (ThT) fluorescence assay confirmed significant association of Poly(I:C) into lysozyme aggregates after 24 h. Data are shown as mean ± SEM (*n* = 3). Statistical significance is indicated as *** *p* < 0.001. (**B**) TEM images show that Lyz-Poly(I:C) aggregates formed longer, denser fibrillar structures after 24 h. Scale bar: 100 nm.

Next, the interactions of lysozyme aggregates with synthetic RNA and formulations with polyinosinic–polycytidylic acid (Poly (I:C)) were prepared and characterized. First, the zeta potential for the combo was determined to provide rationale for the system since incorporation of Poly (I:C) would not occur if there was charge repulsion, and hence to verify that they interacted based on surface charge and electrostatic interactions. Poly (I:C) exhibited a strongly negative zeta potential of −64.467 ± 1.880 mV, confirming its anionic nature (Table 1). Next, the zeta potential of Lyz-Poly (I:C) was found to be −0.934 ± 0.713 mV (Table 1) indicating partial charge neutralization and successful electrostatic interaction. ThT fluorescence analysis revealed significant aggregation for Lyz-Poly (I:C) occurred (*p*-value < 0.001) as shown in (Figure 2A). Additionally, TEM imaging was performed to verify the morphology and likely successful incorporation of Poly (I:C). Lyz-Poly (I:C) aggregates formed longer, denser fibrillar structures than aggregates with lysozyme alone, with a size of around 200 nm (Figure 2B). However, DLS measurements showed a larger average size of 475.875 ± 108.73 nm with higher polydispersity, indicating denser fibrillar structures and aggregates. This increase in size and density, along with the altered zeta potential and ThT signal, provided multiple lines of evidence for the successful incorporation of Poly (I:C) into the lysozyme fibril network.

**Figure 3:**
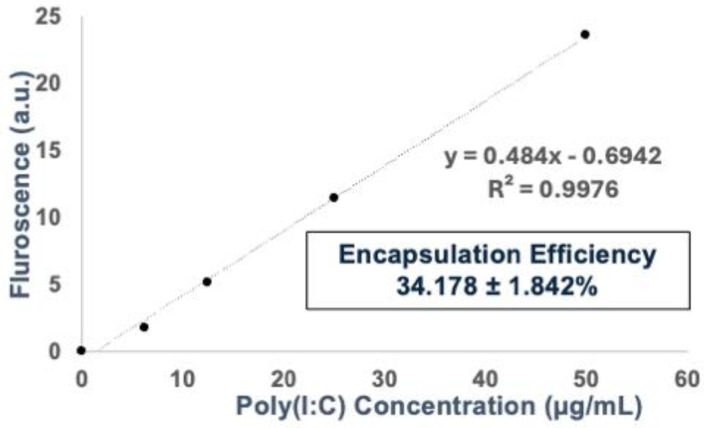
Encapsulation efficiency of Poly (I:C) in lysozyme aggregates using a fluorescence calibration curve. A standard calibration curve was created using Rhodamine-labeled Poly (I:C) at concentration ranging from 0 to 100 μg/mL, measured at 546 nm excitation and 576 nm emission. From the resulting linear equation (y = 0.484x − 0.6942), the encapsulation was determined to be 34.178 ± 1.842%. Data are shown as mean ± SEM (*n* = 3).

Since Lyz-Poly (I:C) showed potential as a delivery depot system, it was further characterized by encapsulation efficiency and release studies for use in later cellular efficacy studies. The encapsulation efficiency of Poly(I:C) in lysozyme was calculated using a calibration curve of Poly (I:C) concentrations from 0 to 100 µg/mL labeled with Rhodamine to generate a fluorescence curve at 546/576 nm excitation/emission. The amount of Poly (I:C) was calculated from the curve (y = 0.484 x−0.6942) (Figure 3), and the encapsulation efficiency for Poly (I:C) was found to be 34.178 ± 1.842% (Table 2). Cumulative release profiles over 24 and 48 h showed that 54.168 ± 3.414% and 67.574 ± 1.273% were released, respectively, suggesting sustained release from the aggregates over time (Table 2). 

**Figure 4:**
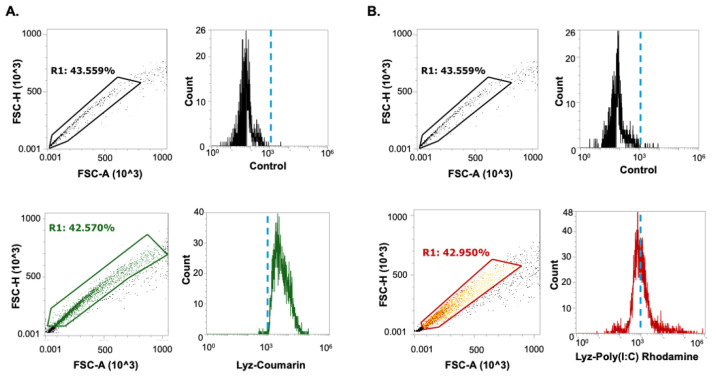
The cellular uptake of amyloid aggregates was investigated using flow cytometry. RAW 264.7 cells were treated with (**A**) lysozyme labeled with Coumarin, or (**B**) Lyz-Poly (I:C) Rhodamine for 30 min, and then uptake was analyzed. Singlet cell population was chosen from FSC-A vs. FSC-H plots, and the uptake was compared to control cells without any treatment. Flow cytometry measurements depict a shift in fluorescence with aggregate treatment, and uptake-positive populations were identified above the 10^3^ intensity threshold.

Flow cytometry was used to quantify uptake of Lyz and Lyz-Poly (I:C) in RAW 264.7 macrophage-like cells after 30 min treatment. Fluorescently labeled aggregates were analyzed using a consistent square gating strategy to define uptake-positive populations above the 10^3^ intensity threshold. Coumarin-labeled Lyz demonstrated high uptake, with 83.971 ± 6.782 % of cells falling within the gated region (Figure 4A). Rhodamine-labeled Lyz-Poly (I:C) had 35.156 ± 4.983 % of the gated cell population fall within the defined window (Figure 4B). Although Lyz-Poly (I:C) was internalized, its lower uptake efficiency may be related to the near-neutral zeta potential (–0.93 mV), which likely reduced electrostatic interaction with the negatively charged cell membrane. Moreover, the reduced surface charge may have promoted agglomeration, limiting bioavailability and cellular dispersion. Nonetheless, both formulations were taken up by cells, indicating the capacity for intracellular delivery.

**Figure 5:**
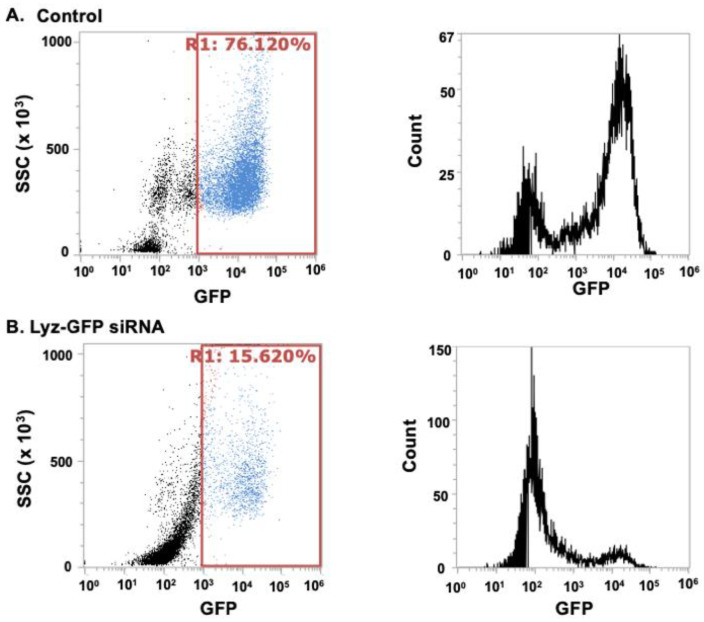
Flow cytometry analysis of GFP knockdown in A549 GFP cells treated with Lyz-siRNA. A549 GFP cells were incubated with Lyz-GFP siRNA for 24 h to assess functional siRNA delivery. GFP expression was measured using flow cytometry. Three independent experiments were carried out. A representative image of one experiment is depicted in the figure showing Lyz-GFP siRNA treatment led to a significant decrease in GFP-positive cells (15.62%) compared to control (76.12%).

**Figure 6:**
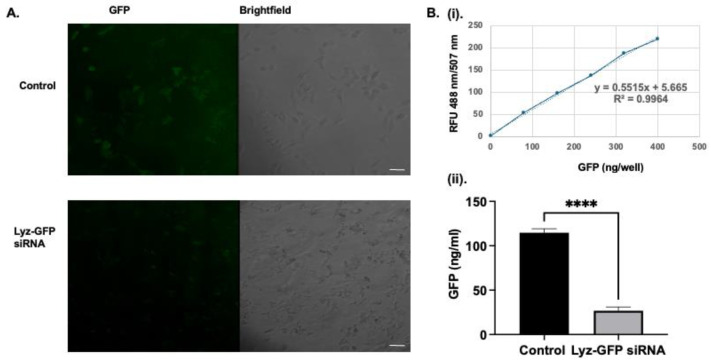
(**A**)**.** Confocal images of GFP knockdown in A549 GFP cells treated with Lyz-GFP siRNA. A549 GFP cells were incubated with Lyz-GFP siRNA for 24 h to assess functional siRNA delivery. GFP expression was imaged with confocal microscopy. Scale bar: 50 µm. (**B**)**.** GFP quantification assay. (**i**)**.** Calibration curve. (**ii**)**.** GFP amount in lysed cells. Control cells show significant amount of GFP compared to cells treated with Lyz-GFP siRNA. Data are shown as mean ± SEM (*n* = 3). Statistical significance is indicated as **** *p* < 0.0001.

Secondarily, to further validate intracellular delivery and assess functional RNA delivery, Lyz was loaded with GFP-targeting siRNA and incubated with A549-GFP cells to determine if there was a decrease in GFP expression, verifying transfection of aggregates. First, the zeta potential of siRNA alone was −19.677 ± 2.172 mV (Table 3), while Lyz-siRNA complexes had a positive charge of 10.257 ± 0.220 mV confirming favorable electrostatic interaction (Table 3). Further, size measurements by DLS resulted in an average size of 377.87 ± 89.47 nm. After verifying size and surface charge and electrostatic interactions, the A549-GFP cell line was treated with Lyz-siRNA and allowed to incubate for 24 h to allow for internalization and gene silencing. GFP expression was subsequently quantified using flow cytometry, employing a gating strategy that defined GFP-expressing cells to the right of the 10^3^ fluorescence intensity threshold based on untreated controls (Figure 5A,B). Following analysis of three independent treatments, cells exposed to Lyz-GFP siRNA displayed 87.41 ± 2.40% of the GFP-positive population relative to the control, indicating substantial knockdown of GFP expression and confirming intracellular delivery of functional siRNA. However, as can be seen from the images, there are excess amyloid aggregates in the extracellular space, indicating the need for further optimization of the dosage of the formulation. To corroborate flow cytometry findings, confocal microscopy was performed. Confocal imaging showed a similar result to flow cytometry, as a significantly diminished cytoplasmic GFP signal was observed compared to the control, as shown in (Figure 6A). Image J 1.54k (Wayne Rasband National Institute of Health, Bethesda, MD, USA) analysis revealed mean fluorescence intensity of 19.441 ± 5.545 and 2.065 ± 0.557 for the cells with no treatment and cells with Lyz-GFP siRNA, respectively. In addition, the effect of Lyz-GFP siRNA was verified by GFP quantification assay. The assay revealed 69.283 ± 2.410 and 8.665 ± 3.731 ng/mL GFP amounts for the cells with no treatment and cells with Lyz-GFP siRNA (Figure 6B). Overall, flow cytometry, confocal imaging, and GFP quantification assay verified the previous cellular uptake study and further demonstrated the potential of lysozyme aggregates for RNA delivery.

**Figure 7:**
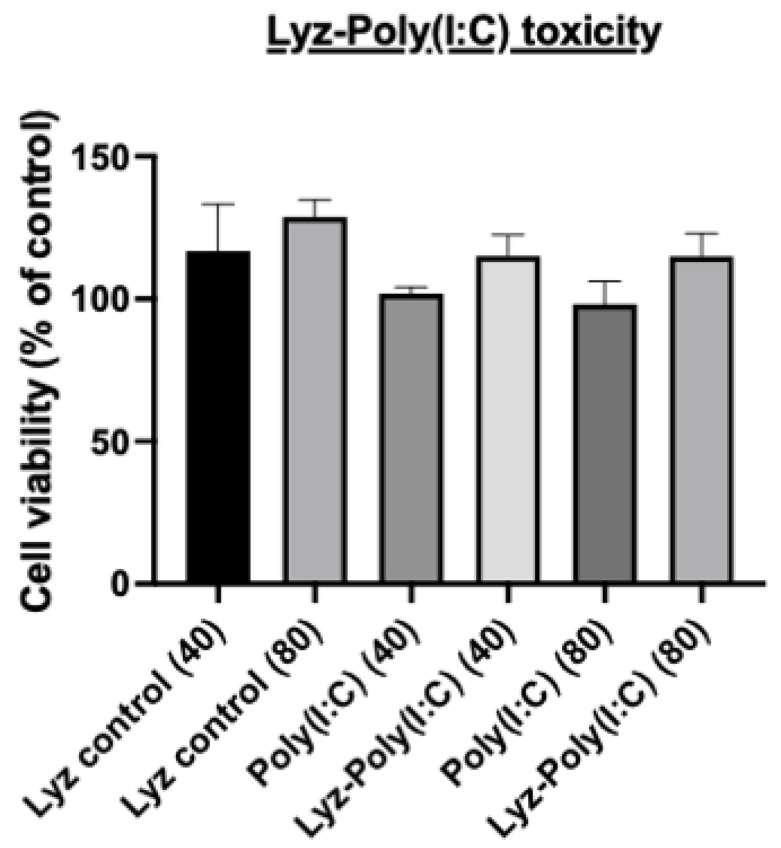
Cytotoxicity assessment of Lyz, Poly (I:C), and Lyz-Poly (I:C) formulation in RAW 264.7 cells. RAW 264.7 macrophages were treated with lysozyme, Poly (I:C), and their combination at concentrations of 40 and 80 μg/mL for 24 h. Cell viability was assessed by the AlamarBlue assay. All treatments maintained cell viability above 90% with no significant differences compared to untreated controls, confirming that the formulations are non-toxic and suitable for subsequent immunological experiments.

Next, the rationale for using Poly (I:C) as a model synthetic dsRNA was verified, as we wanted to determine if the immunostimulatory effects of Poly (I:C) would still be expressed in RAW 264.7 macrophage cells derived from murine leukemia tumors with our Lyz aggregate delivery depot system. First, we determined if there were any cytotoxic effects using an Alamar blue assay. Cells were treated with Lyz alone, Poly (I:C) alone, and Lyz-Poly (I:C) formulations at two concentrations (40 μg/mL and 80 μg/mL) for 24 h. As shown in (Figure 7), none of the treatments induced significant cytotoxicity compared to untreated controls. Across all groups, cell viability remained above 90%, confirming that lysozyme-based aggregates are well tolerated under the tested conditions and appropriate for downstream immune assays.

**Figure 8:**
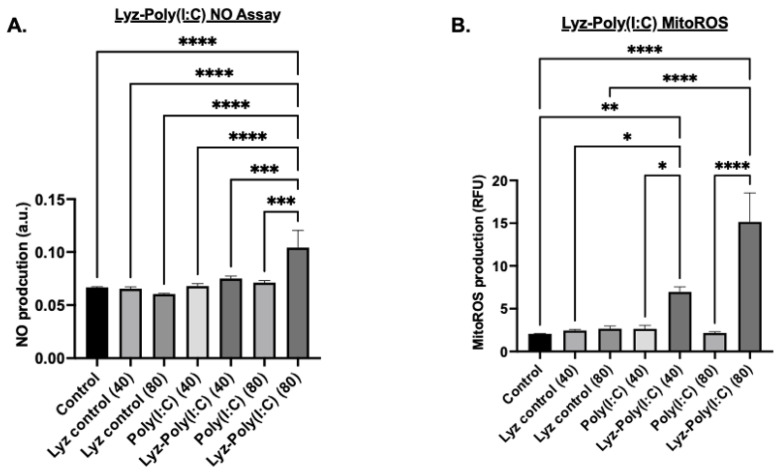
Inflammatory response to Lyz-Poly (I:C) aggregates in RAW 264.7 cells. (**A**) Nitric oxide (NO) production measured in cells treated with lysozyme–Poly (I:C) aggregates, Poly (I:C), and control. Lysozyme–Poly (I:C) aggregates induced the highest NO production compared to other treatments. Data are shown as mean ± SEM (*n* = 3). Statistical significance: *** *p* < 0.001, **** *p* <0.0001. (**B**) Mitochondrial ROS production in cells treated with lysozyme–Poly (I:C) aggregates, Poly (I:C), and control. Lysozyme–Poly (I:C) aggregates showed the highest mitochondrial ROS production. Data are shown as mean ± SEM (*n* = 3). Statistical significance: * *p* < 0.05, ** *p* <0.01, **** *p* < 0.0001.

Additional tests were carried out under the same conditions previously stated to verify if an inflammatory response was occurring. Nitric oxide (NO) production measurements were carried out after 24 h. NO production, a hallmark of macrophage activation and innate immune response, was quantified using the Griess reagent assay with absorbance measured at 540 nm. The results showed that Lyz-Poly (I:C) 80 µg/mL aggregates induced significant NO production compared to all other conditions (*p*-value < 0.0001, Figure 8A). A dose-dependent response was observed, with 80 μg/mL Lyz-Poly (I:C) inducing significantly higher NO levels than the 40 μg/mL formulation (*p* < 0.001, Figure 8A). Notably, Lyz-alone treatments, regardless of concentration, did not elicit measurable NO production, suggesting that immunostimulation was primarily driven by Poly(I:C) and its incorporation into the lysozyme aggregate (Figure 8A). Additionally, to verify that the amyloid aggregates induced an inflammatory response, mitochondrial reactive oxygen species (mitoROS) were measured. Mitochondrial ROS were assessed because they are an additional indicator of cellular stress and inflammatory signaling. The concentration of previous cellular studies was used, and cells were treated with MitoROS 580 working reagent from AAT Bioquest MitoROS assay, and fluorescence was measured at 500/582 nm. As shown in (Figure 8B), Lyz-Poly (I:C) aggregates significantly elevated mitoROS levels in a dose-dependent manner. The 80 μg/mL condition induced the highest level of mitoROS production compared to both Poly (I:C) alone and Lyz alone (*p* < 0.0001), while the 40 μg/mL dose also produced a significant increase over corresponding controls (*p* < 0.01) (Figure 8B). Again, Lyz alone did not significantly increase mitoROS levels, further suggesting that immune activation is a function of Poly (I:C) delivery through the aggregate system. 

**Figure 9:**
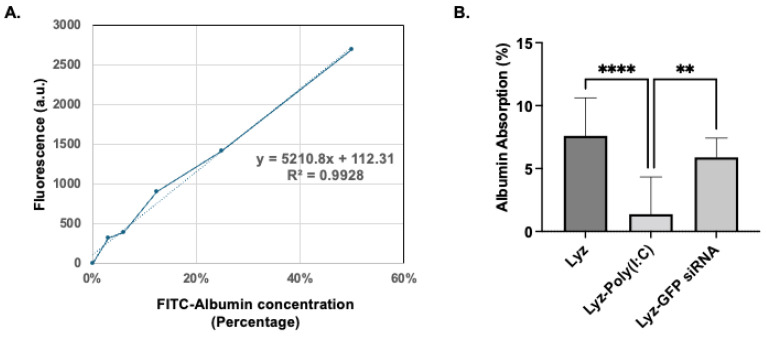
FITC-Albumin protein absorption on lysozyme aggregates. (**A**). Fluorescence calibration curve was created using FITC-Albumin at concentration ranging from 0 to 60% of FITC-Albumin used for the absorption experiment, measured at 495 nm excitation and 520 nm emission. The results of the linear equation were (y = 5210.8x + 112.31), (**B**)**.** The absorption amount was determined for each formulation. Data are shown as mean ± SEM (*n* = 3). Statistical significance: ** *p* < 0.01, **** *p* < 0.0001.

Further, to assess the stability of the amyloids, preliminary protein absorption studies were carried out. Amyloid formulations after incubation with FITC-Albumin for 1 h were tested for their protein absorption. The calibration curve of FITC-Albumin was obtained (Figure 9A), and the amount of protein absorption was determined (Figure 9B). The results show FITC-Albumin absorbance percentages of 7.59 ±1.00, 1.34 ± 0.98, and 5.87 ± 0.51 for Lyz, Lyz-Poly (I:C), and Lyz-GFP siRNA amyloids, respectively. Lyz and Lyz-GFP siRNA amyloids exhibited significantly more absorbance compared to Lyz-Poly (I:C), potentially due to their positive zeta potential. Finally, all these findings from the study highlight the potential of Lyz-based amyloids for the delivery of RNA for a variety of applications. 

## 4. Discussion

This study demonstrates that lysozyme amyloid aggregates are a promising platform for synthetic RNA delivery, offering structural stability, biocompatibility, and effective encapsulation of synthetic nucleic acids such as Poly (I:C). Under acidic conditions, lysozyme formed fibrillar aggregates characterized by ThT fluorescence and confirmed via TEM, aligning with previous findings that establish lysozyme’s utility as a model for functional amyloid systems. ThT fluorescence and TEM imaging verified the formation of characteristic fibrillar structures with lengths ranging from 50 to 100 nm for lysozyme alone and 200–250 nm when complexed with Poly (I:C), indicating a substantial morphological shift upon RNA incorporation. DLS measurements however revealed slightly higher size for the amyloids, indicating potential aggregation formation. However, since DLS is normally used for spherical particles, the fibrillar morphology of the amyloids may not be well represented by the DLS measurements.

Notably, the addition of Poly (I:C) significantly enhanced amyloid formation, as evidenced by elevated ThT fluorescence. This suggests that Poly (I:C) not only binds but may also promote fibril maturation or stabilization. The mechanism of this enhancement could stem from electrostatic interactions between the anionic backbone of Poly (I:C) and the cationic residues of lysozyme. Furthermore, the shift in zeta potential from 18.4 mV Lyz alone to near-neutral –0.93 mV in the Lyz-Poly (I:C) complex supports a charge neutralization effect that may favor aggregation through reduced inter-fibril repulsion. Similar charge-driven aggregation has been reported for other nucleic acid complexes [35].

Encapsulation and release studies further the utility of lysozyme aggregates as delivery depots. With an encapsulation efficiency of approximately 34%, the aggregates demonstrated sustained release of Poly (I:C), with over 67% cumulative release by 48 h. This slow-release behavior is particularly advantageous for immunotherapeutic applications, where prolonged exposure may be necessary to sustain immune activation. The sustained release kinetics are likely driven by the physical entrapment of RNA within the fibrillar network and modulated by charge interactions.

Cellular uptake studies confirmed that both Lyz and Lyz-Poly (I:C) formulations were internalized by RAW 264.7 macrophages, although the uptake was lower for the near-neutral Lyz-Poly (I:C). This may be attributed to diminished electrostatic attraction between the aggregates and negatively charged cellular membranes or increased agglomeration, reducing bioavailability. Further, we verified this by the reduction in GFP fluorescence in A549-GFP cells. Despite lower uptake, functional validation using GFP-targeting siRNA demonstrated more than 85% knockdown in GFP expression in A549-GFP cells, confirming that lysozyme aggregates are not only internalized but also capable of mediating functional RNA delivery. However, the confocal images revealed excess amyloid aggregates in the extracellular space after treatment, indicating the need for further optimization on the concentration, stability, and dosage of the formulation. The ability of lysozyme amyloid aggregates to successfully deliver siRNA and preserve its function confirms that our platform can protect and deliver synthetic RNA intracellularly without compromising bioactivity. These findings support that the inflammatory response observed with Poly (I:C) delivery was a result of intracellular release and recognition, not simply surface-level interactions or aggregate-related effects.

Viral vectors such as adeno-associated virus (AAV) and lentivirus remain the gold standard for transduction efficiency and sustained gene expression. However, they carry risks such as genomic integration and immune reactions [36]. By comparison, lysozyme amyloids offer a non-integrative, transient depot that enables slow, controlled RNA release ideal for applications like vaccine adjuvants or immunotherapies. Although viral vectors can deliver larger payloads at once, our system’s knockdown efficiency is on par with many non-viral carriers reported in the literature [37,38,39].

Importantly, Lyz-Poly (I:C) formulations retained the immunostimulatory properties of Poly (I:C), as indicated by significantly elevated nitric oxide (NO) and mitochondrial ROS production in macrophage cultures. The dose-dependent increase in both markers confirms that the aggregates deliver biologically active RNA that triggers innate immune signaling. Notably, lysozyme aggregates alone failed to elicit similar responses, indicating that immune stimulation is specifically driven by the RNA cargo rather than the protein aggregates. These are compared to many cationic polymer nanoparticles, which can cause toxicity and unwanted inflammation due to membrane disruption [40]. Our data show lysozyme amyloids are safer as they do not trigger nitric oxide or ROS on their own. The immune response comes solely from the RNA payload, which solves a big problem seen with many polymer carriers [41]. Finally, to realize the translational potential, stability and protein absorption properties need to be fully investigated. Our preliminary findings indicate minimal absorption for the near-neutral Lyz-Poly (I:C) compared to Lyz or Lyz-GFP siRNA. This may be attributed to the interaction between the positively charged aggregates and negatively charged Albumin.

Overall, these findings highlight the potential of lysozyme amyloid aggregates as multifunctional RNA carriers that can protect, deliver, and release RNA therapeutics while preserving their immunological activity. The aggregates exhibit key hallmarks of an ideal delivery system: structural integrity, favorable encapsulation and release profiles, cellular uptake capability, immune modulation, and biocompatibility. Thus, lysozyme amyloids have added advantages compared to other non-viral drug delivery systems which have been shown to impart toxicity and immune responses [39,42]. Future studies should focus on elucidating the precise molecular mechanisms by which RNA modulates amyloid assembly, optimizing surface charge and aggregate size to improve cellular uptake, and evaluating efficacy in in vivo models of infection, cancer, or inflammation. Collectively, our findings support lysozyme amyloids as non-viral RNA carriers. The expanding utility of amyloid-based materials in nanomedicine underscores the translational promise of lysozyme aggregates for RNA-based immunotherapies and gene-silencing strategies.

## Figures and Tables

**Table 1:** Zeta potential of lysozyme (Lyz) aggregates, Poly(I:C), and Lyz-Poly(I:C) complexes.

Formulation	Zeta Potential (mV)
Lyz	18.433 ± 0.578
Poly (I:C)	−64.467 ± 1.880
Lyz-Poly (I:C)	−0.934 ± 0.713

**Table 2:** Poly(I:C) encapsulation and release.

Formulation	Encapsulation Efficiency (%)	Drug Release (%)	
Lyz-Poly (I:C)	34.178 ± 1.842	24 h	48 h
		54.168 ± 3.414	67.574 ± 1.273

**Table 3:** Zeta potential of siRNA and Lyz-siRNA complexes.

Formulation	Zeta Potential (mv)
siRNA	−19.677 ± 2.172
Lyz-GFP siRNA	10.257 ± 0.220

## Data Availability

All the data related to the results of the article will be made available by the authors, without any restrictions.
